# The clinical and molecular landscape of congenital myasthenic syndromes in Austria: a nationwide study

**DOI:** 10.1007/s00415-022-11440-0

**Published:** 2022-10-29

**Authors:** Martin Krenn, Merve Sener, Jakob Rath, Gudrun Zulehner, Omar Keritam, Matias Wagner, Franco Laccone, Stephan Iglseder, Sonja Marte, Manuela Baumgartner, Astrid Eisenkölbl, Christian Liechtenstein, Sabine Rudnik, Stefan Quasthoff, Susanne Grinzinger, Johannes Spenger, Saskia B. Wortmann, Wolfgang N. Löscher, Fritz Zimprich, Anna Kellersmann, Mika Rappold, Günther Bernert, Michael Freilinger, Hakan Cetin

**Affiliations:** 1 Department of Neurology, Medical University of Vienna, Vienna, Austria; 2 Institute of Human Genetics, Technical University of Munich, Munich, Germany; 3 Institute for Neurogenomics, Helmholtz Center Munich, Munich, Germany; 4 Institute of Medical Genetics, Medical University of Vienna, Vienna, Austria; 5 Department of Neurology, Krankenhaus Barmherzige Brüder, Linz, Austria; 6 Neurologie Montfort, Feldkirch, Austria; 7 Department of Neuropaediatrics, Hospital Barmherzige Schwestern Linz, Linz, Austria; 8 Department of Paediatrics and Adolescent Medicine, Johannes Kepler University Linz, Linz, Austria; 9 Department of Paediatrics and Adolescent Medicine, Villach Regional Hospital, Villach, Austria; 10 Institute of Human Genetics, Medical University of Innsbruck, Innsbruck, Austria; 11 Department of Neurology, Medical University of Graz, Graz, Austria; 12 Department of Neurology, Salzburger Landeskliniken, Paracelsus Medical University, Salzburg, Austria; 13 University Children’s Hospital, Paracelsus Medical University, Salzburg, Austria; 14 Amalia Children’s Hospital, Radboudumc, Nijmegen, The Netherlands; 15 Department of Neurology, Medical University of Innsbruck, Innsbruck, Austria; 16 Department of Pediatrics, Klinik Favoriten, Vienna, Austria; 17 Department of Pediatrics and Adolescent Medicine, Medical University of Vienna, Vienna, Austria

**Keywords:** Congenital myasthenic syndrome, Myasthenia, *CHRNE*, Austria

## Abstract

**Background:**

Congenital myasthenic syndromes (CMS) are a heterogeneous group of disorders caused by genetic defects resulting in impaired neuromuscular transmission. Although effective treatments are available, CMS is probably underdiagnosed, and systematic clinico-genetic investigations are warranted.

**Methods:**

We used a nationwide approach to collect Austrian patients with genetically confirmed CMS. We provide a clinical and molecular characterization of this cohort and aimed to ascertain the current frequency of CMS in Austria.

**Results:**

Twenty-eight cases with genetically confirmed CMS were identified, corresponding to an overall prevalence of 3.1 per million (95% CI 2.0–4.3) in Austria. The most frequent genetic etiology was *CHRNE* (*n* = 13), accounting for 46.4% of the cohort. Within this subgroup, the variant c.1327del, p.(Glu443Lysfs*64) was detected in nine individuals. Moreover, causative variants were found in *DOK7* (*n* = 4), *RAPSN* (*n* = 3), *COLQ* (*n* = 2), *GMPPB* (*n* = 2), *CHAT* (*n* = 1), *COL13A1* (*n* = 1), *MUSK* (*n* = 1) and *AGRN* (*n* = 1). Clinical onset within the first year of life was reported in one half of the patients. Across all subtypes, the most common symptoms were ptosis (85.7%), lower limb (67.9%), upper limb (60.7%) and facial weakness (60.7%). The majority of patients (96.4%) received specific treatment, including acetylcholinesterase inhibitors in 20, adrenergic agonists in 11 and 3,4-diaminopyridine in nine patients.

**Conclusions:**

Our study presents the first systematic characterization of individuals with CMS in Austria, providing prevalence estimates and genotype–phenotype correlations that may help to improve the diagnostic approach and patient management.

**Supplementary Information:**

The online version contains supplementary material available at 10.1007/s00415-022-11440-0.

## Introduction

Congenital myasthenic syndromes (CMS) encompass a heterogeneous group of inherited disorders caused by genetic defects that result in impaired signal transmission at the neuromuscular junction (NMJ) [1]. While early-onset fatigable muscle weakness is considered the clinical hallmark of CMS, the phenotypic spectrum may also include other organ manifestations, and symptom severity as well as age of onset are highly variable between genetic subtypes. With the rapid advancements of next-generation sequencing (NGS) technology over the past decade, the number of known molecular defects has constantly grown, and more than 30 underlying monogenic disease genes encoding presynaptic, synaptic and postsynaptic components of the NMJ have been identified to date [2].

CMS are rare with only few prevalence estimates in the literature, ranging between 1.8 per million total population and 22.2 per million children [3, 4]. Epidemiological studies, however, probably underestimate the real prevalence of CMS due to the lack of large disease registries, limited access to NGS-based genetic testing in some countries and the misclassification of CMS as (seronegative) autoimmune myasthenia gravis [5]. Nonetheless, the early identification of underlying genetic defects is crucial in the light of various effective treatment options that are already available for most CMS forms [6].

In our study, we aimed to systematically describe the clinical and molecular spectrum of CMS in Austria. We used a nationwide approach to recruit patients from both pediatric and adult neuromuscular centers and, for the first time, provide prevalence estimates for CMS in Austria.

## Patients and methods

### Patient ascertainment and ethics approval

This retrospective, nationwide cohort study included all patients with genetically confirmed CMS (i.e., related to genes previously associated with CMS) who were treated in one of the Austrian pediatric or adult neuromuscular centers between 01/01/2000 and 31/12/2021. The ethics committee of the Medical University of Vienna approved this study prior to the nationwide acquisition of clinical and genetic patient data (EK: 2065/2018). The study was performed in accordance with the ethical standards laid down in the 1964 Declaration of Helsinki and its later amendments.

### Data collection

Collaborating centers were contacted via e-mail and invited to complete a pseudonymized case report form including demographic, clinical and genetic details. All genetic variants were re-analyzed, and only pathogenic and likely pathogenic variants according to the standards of the American College of Medical Genetics and Genomics (ACMG) were eligible for inclusion in the study, if the variants were compatible with the expected mode of inheritance [7]. Reported variants were submitted to the Global Variome shared Leiden Open-source Variation Database (LOVD, https://databases.lovd.nl) [8]. Given the multicentric nature of this study and the application of genetic tests in different laboratories, applied gene panels, exome and genome analyses may have encompassed different sets of genes with variable coverage. These different NGS approaches were pooled and analyzed together in this study.

### Data analysis

The prevalence of CMS in Austria was estimated based on population data (2020) extracted from “Statistik Austria” (https://www.statistik.at), with 95% confidence intervals (CI) calculated assuming a Poisson distribution. Data were analyzed by SPSS version 28 (IBM Corp., Armonk, NY, USA) using descriptive measures. Figures were created using Prism, version 9.1.0 (GraphPad Software Inc., San Diego, CA, USA).

## Results

### Demographic and clinical characteristics

Twenty-eight individuals from 25 different families with genetically confirmed CMS were identified through our nationwide patient recruitment, corresponding to an overall prevalence of 3.1 per million (95% CI 2.0–4.3) in Austria. The calculated prevalence in the pediatric population (< 19 years) was 10.5 per million (95% CI 5.6–15.3). In our study cohort, females accounted for 64.3%. One half of the patients experienced first symptoms just after birth or during the first year of life (50%). Disease onset in adolescence (13–18 years) and adulthood (> 18 years), by contrast, was very rare, with only one case represented in each age group (both associated with biallelic variants in *GMPPB*) (Table 1).

**Table 1:** Demographic and clinical characteristics of total CMS cohort and patients with causative variants in *CHRNE*

	Total cohort (*n* = 28)	*CHRNE* (*n* = 13)
Sex, female (%)	18 (64.3)	8 (61.5)
Positive family history (%)	10 (35.7)	7 (53.8)
Age group at onset (%)^a^		
At birth	7 (26.9)	3 (23.1)
Infancy (< 1 year)	6 (23.1)	4 (30.8)
Childhood (1–13 years)	11 (42.3)	6 (46.2)
Adolescence (13–18 years)	1 (3.8)	0 (0)
Adulthood (> 18 years)	1 (3.8)	0 (0)
Genetic analysis leading to molecular diagnosis (%)^b^		
Single gene testing	6 (27.3)	2 (22.2)
Next-generation sequencing	16 (72.7)	7 (77.8)
Repetitive nerve stimulation (%)^c^		
Decrement	5 (62.5)	3 (50.0)
Normal	3 (37.5)	3 (50.0)
Mobility (%)^d^		
Independent	20 (76.9)	12 (92.3)
Walking aid/rollator	1 (3.8)	0 (0)
Wheelchair	5 (19.2)	1 (7.7)
Medical treatment (%)		
Pyridostigmine	20 (71.4)	13 (100)
Adrenergic agonists	11 (39.3)	1 (7.7)
Amifampridine	9 (32.1)	4 (30.8)
No treatment reported	1 (3.6)	0 (0)

^a^Available for 26 individuals of the total cohort and for all 13 individuals of the *CHRNE* cohort

^b^Available for 22 individuals of the total cohort and 9 individuals of the *CHNRE* cohort

^c^Available for eight individuals of the total cohort and 6 individuals of the *CHNRE* cohort

^d^Available for 26 individuals of the total cohort and all 13 individuals of the *CHNRE* cohort

**Fig. 1:**
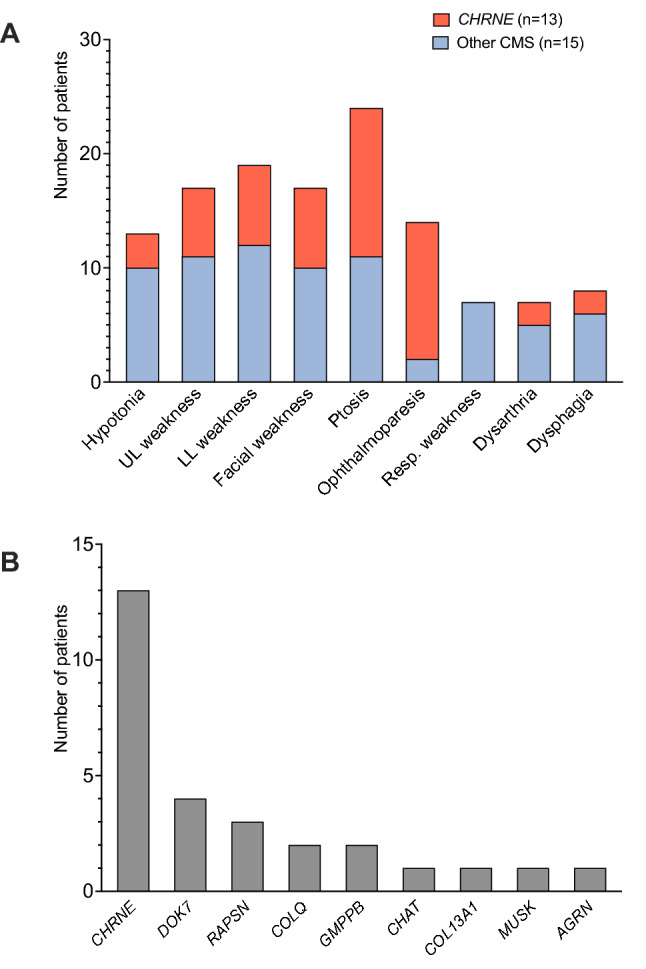
Clinical and genetic spectrum of patients with CMS in Austria. **A** Frequency of phenotypic features compared between patients with *CHRNE*-associated CMS and the remaining molecular etiologies. The *CHRNE* subgroup is predominantly characterized by ocular features (ophthalmoparesis/ophthalmoplegia and ptosis) and the absence of respiratory symptoms, while limb and bulbar weakness was more commonly observed in patients with other CMS forms. **B** The identification of nine different molecular etiologies within the cohort reflects the remarkable genetic heterogeneity of CMS. *CHRNE* and *DOK7* are the two most commonly mutated genes together accounting for more than 60% of the whole cohort

The most commonly observed clinical features across all genetic subtypes were ptosis (85.7%), lower limb (67.9%), upper limb (60.7%) and facial weakness (60.7%). Ophthalmoparesis/ophthalmoplegia was present in 50.0% of the cohort and the dominant symptom in patients with causative *CHNRE* variants (Fig. 1A). Apart from neuromuscular symptoms, further manifestations included scoliosis (25%), facial dysmorphism (14.3%) and gastrointestinal symptoms (10.7%). One patient (with *MUSK*-related CMS) was very severely affected requiring percutaneous endoscopic gastrostomy and invasive ventilation. Another patient with *AGRN*-related CMS required non-invasive ventilation. Lung function tests were available for 10 individuals, showing a mean forced vital capacity (FVC) of 71.4% (total range: 34–94%).

A pathological decrement on repetitive nerve stimulation was reported in five patients and elevated serum creatine kinase (CK) activity in two patients (both with pathogenic *GMPPB* variants).

### Treatment-related aspects

The vast majority of the cohort (96.4%) had received CMS-specific medical treatment at any time point, with acetylcholinesterase inhibitors (pyridostigmine, dosage range: 1.25–7.5 mg/kg/d) used in 20 patients (71.4%), and adrenergic agonists (salbutamol, dosage range: 0.1–1.39 mg/kg/d) and 3,4-diaminopyridine (3,4-DAP, dosage range: 0.1–1.25 mg/kg/d) used in 11 (39.3%) and nine patients (32.1%), respectively. A significant proportion of patients (39.3%) had multiple treatments, either used sequentially or in combination. General clinical improvement attributable to medication was reported in two thirds (66.7%) and specific improvement of ocular symptoms in 37% of treated patients.

All 13 patients with *CHRNE*-associated CMS received targeted medical treatment. Pyridostigmine was used in all patients, while 3,4-DAP was used in four and salbutamol only in one. In addition to medication, ptosis surgery (blepharoplasty) was performed in two individuals with *CHRNE*-related CMS.

The second most common genetic etiology in our series was *DOK7*, which shows an excellent response to treatment with salbutamol [9, 10]. All four *DOK7* cases reported in this study received adrenergic agonists, which resulted in marked clinical improvement in three of them.

Summarized case report files of the 28 individuals with CMS including clinical and demographic details are provided as supplementary information.

### Genetic findings

Twenty-four different pathogenic or likely pathogenic (i.e., diagnostic) variants according to ACMG standards were identified in nine different CMS-related genes (Fig. 1B and Table 2), with variants in the most commonly affected genes *CHRNE* and *DOK7* accounting for 46.4% and 14.3% of the cohort, respectively. All genetically solved cases displayed an autosomal recessive inheritance pattern with homozygous or compound heterozygous variants in known disease genes. The spectrum of detected variants included 14 different missense variants, five frameshift variants, two canonical splice-site variants, one nonsense variant, one near-splice variant and one copy number variant (microdeletion). Overall, more molecular diagnoses could be established by NGS-based approaches than by single gene sequencing (16 vs. six cases, data missing in six cases).

**Table 2:** List of causative genetic variants identified in 28 Austrian patients with CMS

Patient ID	Gene (transcript)	Variant [zygosity]	ACMG criteria fulfilled	ACMG classification
1	*RAPSN* (NM_005055.5)	c.264C>A, p.(Asn88Lys) [homozygous]	PS1, PS3, PM1, PM2, PM3, PP3	Pathogenic
2	*CHRNE* (NM_000080.4)	c.1327del, p.(Glu443Lysfs*64) [homozygous]	PVS1, PM2, PM3, PP3	Pathogenic
3	*CHAT* (NM_020549.5)	c.1669G>A, p.(Ala557Thr) [heterozygous]	PS1, PM2, PM3, PP3	Likely pathogenic
		c.1267G>A, p.(Asp423Asn) [heterozygous]	PM1, PM2, PM3, PP3	Likely pathogenic
4	*DOK7* (NM_173660.5)	c.1388_1446del, p.(Glu463Glyfs*36) [heterozygous]	PVS1, PM2, PM3, PP3	Pathogenic
		c.1124_1127dup, p.(Ala378Serfs*30) [heterozygous]	PVS1, PM2, PM3, PP3	Pathogenic
5	*CHRNE* (NM_000080.4)	c.1327del, p.(Glu443Lysfs*64) [homozygous]	PVS1, PM2, PM3, PP3	Pathogenic
6	*CHRNE* (NM_000080.4)	c.1327del, p.(Glu443Lysfs*64) [homozygous]	PVS1, PM2, PM3, PP3	Pathogenic
7	*GMPPB* (NM_021971.4)	c.79G>C, p.(Asp27His) [heterozygous]	PS1, PM1, PM2, PM3, PP3	Likely pathogenic
		c.848G>C, p.(Gly283Ala) [heterozygous]	PM1, PM2, PM3, PP3	Likely pathogenic
8	*DOK7* (NM_173660.5)	c.439dup, p.(Ala147Glyfs*10) [heterozygous]	PVS1, PM2, PM3, PP3	Pathogenic
		c.1124_1127dup, p.(Ala378Serfs*30) [heterozygous]	PVS1, PM2, PM3, PP3	Pathogenic
9	*CHRNE* (NM_000080.4)	c.1327del, p.(Glu443Lysfs*64) [homozygous]	PVS1, PM2, PM3, PP3	Pathogenic
10	*CHRNE* (NM_000080.4)	c.1327del, p.(Glu443Lysfs*64) [homozygous]	PVS1, PM2, PM3, PP3	Pathogenic
11	*AGRN* (NM_198576.4)	c.3419T>C, p.(Leu1140Pro) [heterozygous]	PM1, PM2, PM3	Likely pathogenic
		chr1.hg19: g.896373_975646del [heterozygous]	PVS1, PM2, PM3	Pathogenic
12	*COLQ* (NM_005677.3)	c.965T>G, p.(Val322Gly) [heterozygous]	PM1, PM2, PP3, PP4	Likely pathogenic
		c.1026C>G, p.(Asp342Glu) [heterozygous]	PM1, PM2, PP3, PP4	Likely pathogenic
13	*CHRNE* (NM_000080.4)	c.1327del, p.(Glu443Lysfs*64) [homozygous]	PVS1, PM2, PM3, PP3	Pathogenic
14	*CHRNE* (NM_000080.4)	c.1327del, p.(Glu443Lysfs*64) [homozygous]	PVS1, PM2, PM3, PP3	Pathogenic
15	*DOK7* (NM_173660.5)	c.1124_1127dup, p.(Ala378Serfs*30) [heterozygous]	PVS1, PM2, PM3, PP3	Pathogenic
		c.472C>T, p.(Arg158Trp) [heterozygous]	PM1, PM2, PM3	Likely pathogenic
16	*CHRNE* (NM_000080.4)	c.1327del, p.(Glu443Lysfs*64) [homozygous]	PVS1, PM2, PM3, PP3	Pathogenic
17	*DOK7* (NM_173660.5)	c.1124_1127dup, p.(Ala378Serfs*30) [heterozygous]	PVS1, PM2, PM3, PP3	Pathogenic
		c.1378dup, p.(Gln460Profs*59) [heterozygous]	PVS1, PM2, PM3, PP3	Pathogenic
18	*GMPPB* (NM_021971.4)	c.79G>C, p.(Asp27His) [heterozygous]	PS1, PM1, PM2, PM3, PP3	Likely pathogenic
		c.860G>A, p.(Arg287Gln) [heterozygous]	PS1, PM1, PM2, PM3	Pathogenic
19	*CHRNE* (NM_000080.4)	c.1327del, p.(Glu443Lysfs*64) [homozygous]	PVS1, PM2, PM3, PP3	Pathogenic
20	*MUSK* (NM_005592.4)	c.1744G>A, p.(Gly582Arg) [heterozygous]	PM1, PM2, PM3, PP3, PP4	Likely pathogenic
		c.698G>A, p.(Cys233Tyr) [heterozygous]	PM1, PM2, PM3, PP3, PP4	Likely pathogenic
21	*CHRNE* (NM_000080.4)	c.1326+1G>A, p? [homozygous]	PVS1, PM2, PM3, PP1, PP3	Pathogenic
22	*CHRNE* (NM_000080.4)	c.1326+1G>A, p? [homozygous]	PVS1, PM2, PM3, PP1, PP3	Pathogenic
23	*COLQ* (NM_005677.4)	c.943C>T, p.(Arg315*) [homozygous]	PVS1, PM2, PM3, PP3	Pathogenic
24	*COL13A1* (NM_001368882.1)	c.715-1G>A, p? [homozygous]	PVS1, PM2, PM3, PP3	Pathogenic
25	*RAPSN* (NM_005055.5)	c.193-15C>A, p? [heterozygous]	PS3, PM2, PM3, PP1, PP3	Likely pathogenic
		c.264C>A, p.(Asn88Lys) [heterozygous]	PS1, PS3, PM1, PM2, PM3, PP1, PP3	Pathogenic
26	*RAPSN* (NM_005055.5)	c.193-15C>A, p? [heterozygous]	PS3, PM2, PM3, PP1, PP3	Likely pathogenic
		c.264C>A, p.(Asn88Lys) [heterozygous]	PS1, PS3, PM1, PM2, PM3, PP1, PP3	Pathogenic
27	*CHRNE* (NM_000080.4)	c.872C>T, p.(Ala291Val) [heterozygous]	PM1, PM2, PM3, PP1, PP3	Likely pathogenic
		c.103T>C, p.(Tyr35His) [heterozygous]	PM1, PM2, PM3, PP1, PP3	Likely pathogenic
28	*CHRNE* (NM_000080.4)	c.872C>T, p.(Ala291Val) [heterozygous]	PM1, PM2, PM3, PP1, PP3	Likely pathogenic
		c.103T>C, p.(Tyr35His) [heterozygous]	PM1, PM2, PM3, PP1, PP3	Likely pathogenic

*ACMG* American College of Medical Genetics and Genomics; *PM* pathogenic moderate; *PP* pathogenic supporting; *PS* pathogenic strong; *PVS* pathogenic very strong

### *CHRNE*-associated CMS

The majority of *CHRNE*-associated CMS cases (9/13 patients, 69.2%) were caused by the truncating founder variant c.1327del, p.(Glu443Lysfs*64), either in a homozygous or a compound heterozygous carrier state.

Similar to our findings in the total cohort, around one half of the patients with *CHRNE*-associated CMS (53.8%) experienced symptom onset during the first year of life, and all patients before adolescence. Disease severity was generally milder in this subgroup with predominantly ocular symptoms including ophthalmoparesis in 12/13 patients and ptosis in 13/13 patients. By contrast, 12/13 patients (92.3%) in this subgroup were still able to walk independently at the last follow-up visit (as compared to 73.3% in the remainder of the cohort), and respiratory weakness was not observed at all in patients with *CHRNE*-CMS. Only one patient carrying the abovementioned founder variant was wheelchair-dependent.

## Discussion

In this study, we present the first clinical and molecular characterization of pediatric and adult patients with genetically confirmed CMS in Austria who were ascertained through a systematic, nationwide approach. Such collaborative efforts are generally important, as they have the potential to raise awareness for orphan diseases and may therefore improve diagnostic and therapeutic patient services in the long run.

Patient recruitment covered all specialized pediatric and adult neuromuscular centers in Austria and thus enabled us to perform population-based calculations on CMS epidemiology, yielding a prevalence of 3.1 per million in the total population and 10.5 per million in the pediatric population. These figures were comparable to the CMS prevalence of 9.2 per million children in the United Kingdom [11].

However, it is likely that existing epidemiological data rather underestimate the real number of affected patients. First, this may partly be explained by the fact that disease gene discovery is an ongoing process with novel molecular etiologies yet to be identified. Second, there is emerging evidence that hereditary myasthenic syndromes are prone to misdiagnoses and may falsely be classified as (seronegative) autoimmune myasthenia gravis and even be treated with immunosuppressant drugs [5, 12]. All the more so, a precise and early molecular diagnosis is of paramount importance to avoid long diagnostic delays and unnecessary treatments with potential side effects. Third, the identification of CMS cases may also depend on specific conventions in different countries and may be limited by a restricted access to NGS applications. It has already been demonstrated that NGS yields higher diagnostic rates in heterogeneous neuromuscular disorders than sequential gene-by-gene testing [13–15].

Several nationwide studies have already been performed to delineate the genetic spectrum of CMS, and the findings indicate a variable distribution of molecular etiologies and specific mutations between ethnicities and different geographic regions [3, 4, 11, 16–20]. In accordance with data from other European countries, *CHRNE* also represented the most frequently mutated gene in our cohort [3, 4, 11]. Among patients with *CHRNE*-related CMS, the largest part could be attributed to the founder variant c.1327del, p.(Glu443Lysfs*64), which has formerly been referred to as epsilon1267delG. Originating from the Romani (Gypsy) people, this specific variant is particularly frequent in Europe [21]. In line with previous reports, the clinical presentation associated with *CHRNE* variants is heterogeneous and comparably mild, mainly characterized by ophthalmoparesis, which can be helpful to guide genetic testing [22]. Specific physiological features of extraocular muscles such as exceptionally high motor neuron firing rates together with the accumulation of acetylcholine receptors in desensitized states have been suggested to underlie ophthalmoparesis in these patients [23]. In our cohort, over 90% of patients with variants in *CHRNE* had marked limitation of ocular movements but were still able to walk unaided. Patients with variants in *CHRNE* tend to respond favorably to adrenergic agonists [24]. Nonetheless, only one patient in our study was treated with salbutamol, suggesting that available and well-tolerated therapies are still underutilized in these patients.

It is also worthy of note that our study includes a previously unreported individual with *MUSK*-related CMS, a very rare genetic subtype with only a few patients reported to date. It has already been suggested that the phenotypic spectrum associated with *MUSK* variants is very broad, ranging from a mild, fatigable limb-girdle weakness to a severe neonatal syndrome with refractory respiratory failure [25, 26]. Our presented case with *MUSK*-related CMS had a severe phenotype with an onset just after birth, requiring invasive ventilation. However, salbutamol treatment was effective, remarkably improving motor function and reducing the effort of mechanical ventilation.

Only two of the 28 patients in our cohort had their first symptoms reported in adolescence or adulthood. Although clinically heterogeneous, it is well established that the majority of CMS patients have an early onset of disease (in infancy or childhood) [27]. Interestingly, the only two patients in our cohort with an onset beyond childhood carried causative variants in *GMPPB*, a gene that has already been associated with late-onset CMS and limb-girdle muscular dystrophy [28, 29]. In accordance with previous reports, these were also the only patients with significantly elevated CK activity levels [30]. This genetic etiology (together with other genetic defects of glycosylation) should therefore be taken into particular consideration in adult patients presenting with a limb-girdle pattern of myasthenic weakness and significant hyperCKemia [31].

In conclusion, our study provides the first comprehensive clinical and genetic characterization of patients with CMS in Austria. *CHRNE* was the most commonly mutated gene and associated with severe ophthalmoparesis in most patients, which can help to guide diagnosis. In addition, the presented data may be useful to improve diagnostic and therapeutic services for patients and foster collaborative research on this rare and treatable condition.

## Supplementary Information

Below is the link to the electronic supplementary material.

Supplementary file1 (XLSX 15 KB)

## Data Availability

Anonymized data not published in this article will be made available by request from the corresponding author.
